# Proteomic Analyses Reveal Higher Levels of Neutrophil Activation in Men Than in Women With Systemic Lupus Erythematosus

**DOI:** 10.3389/fimmu.2022.911997

**Published:** 2022-06-21

**Authors:** Ming-long Cai, Lan Gui, He Huang, Yu-kun Zhang, Li Zhang, Zhu Chen, Yu-jun Sheng

**Affiliations:** ^1^ Department of Rheumatology and Immunology, the First Affiliated Hospital of USTC, Division of Life Sciences and Medicine, University of Science and Technology of China, Hefei, China; ^2^ Institute of Dermatology and Department of Dermatology of the First Affiliated Hospital, Anhui Medical University, Hefei, China

**Keywords:** systemic lupus erythematosus, neutrophil activation, gender, heterogeneity, proteomics

## Abstract

**Objective:**

Systemic Lupus Erythematosus (SLE) is a systemic autoimmune disease that displays a significant gender difference in terms of incidence and severity. However, the underlying mechanisms accounting for sexual dimorphism remain unclear. The aim of this work was to reveal the heterogeneity in the pathogenesis of SLE between male and female patients.

**Methods:**

PBMC were collected from 15 patients with SLE (7 males, 8 females) and 15 age-matched healthy controls (7 males, 8 females) for proteomic analysis. The proteins of interest were validated in independent samples (6 male SLE, 6 female SLE). Biomarkers for neutrophil activation (calprotectin), neutrophil extracellular traps (cell-free DNA and elastase), and reactive oxygen species (glutathione) were measured, using enzyme-linked immunosorbent assay, in plasma obtained from 52 individuals.

**Results:**

Enrichment analysis of proteomic data revealed that type I interferon signaling and neutrophil activation networks mapped to both male and female SLE, while male SLE has a higher level of neutrophil activation compared with female SLE. Western blot validated that PGAM1, BST2, and SERPINB10 involved in neutrophil activation are more abundant in male SLE than in female SLE. Moreover, biomarkers of neutrophil activation and reactive oxygen species were increased in male SLE compared with female SLE.

**Conclusion:**

Type I interferon activation is a common signature in both male and female SLE, while neutrophil activation is more prominent in male SLE compared with female SLE. Our findings define gender heterogeneity in the pathogenesis of SLE and may facilitate the development of gender-specific treatments.

## Introduction

Systemic Lupus Erythematosus (SLE) is a complex disease characterized by abnormal activation of immune cells, production of autoantibody, immune complex deposition as well as multiple organs damage (1). The global prevalence of SLE ranges from 13 to 7,713.5 per 100,000 persons, and there are great differences among different races (2). In China, it generally affects 38.6 in every 100000 individuals (3). Emerging data show that the prevalence of SLE is rising over time, and now SLE is one of the leading causes of death in young women (2, 4).

The pathogenesis of SLE is not fully understood. Genetic, environmental, and hormonal factors all contribute to disease risk (5). Genetic studies (especially genome-wide association studies) have reported approximately 180 susceptibility loci, including those genes involved in type I interferon (IFN) activation, lymphocyte activation, and innate and adaptive immune response (6). Other omics studies also made great progress in understanding SLE pathogenesis. For instance, gene expression studies have revealed key biological pathways including IFN signaling and neutrophil activation (7–9). However, these studies were mainly focused on transcriptomic profiles with little known about proteome modulation and protein function. Previously studies have shown that in many cases, transcript levels are insufficient to predict protein levels (10) and the average correlation between mRNA and protein expression is below 0.5 (11–13). Therefore, studies at the proteomic level will be crucial and have the potential to reveal new disease pathogenesis absent from transcriptomics data.

SLE has been reported to be highly heterogeneous in terms of gender. The disease is particularly prevalent in women of childbearing age, with a male-to-female incidence ratio of about 1:9. In contrast, men with SLE present with a more severe form of the disease than women in terms of clinical manifestations and prognosis. Studies have shown that male SLE has a more aggressive clinical course with rapid accrual of organ damage (i.e. kidneys, cardiovascular and neuropsychiatric systems), resulting in a poorer prognosis and lower survival rate compared with female SLE (14–16). However, the pathophysiological mechanisms that account for sexual dimorphism are still unclear. In the present study, we performed a whole proteomic profile in male and female SLE to reveal gender heterogeneity in the pathogenesis of SLE. Our study provides new molecular mechanisms in male SLE and may help to develop gender-specific treatment and management.

## Materials and Methods

### Sample Cohorts

Patients with SLE (7 males, 8 females; cohort 1) and age- and sex-matched healthy controls (7 males, 8 females; cohort 1) were recruited from The First Affiliated Hospital of Anhui Medical University. Clinical information, including sex, age, autoantibody, and proteinuria were retrieved from the medical records by at least two investigators. Disease activity was recorded using the systemic lupus erythematosus disease activity index (SLEDAI) score. One cohort (6 male SLE, 6 female SLE; cohort 2) was used for western blot, and one cohort (9 male SLE, 35 female SLE, 4 male HC, 4 female HC; cohort 3) was used for plasma biomarker assessment, also recruited from The First Affiliated Hospital of Anhui Medical University. This study was approved by the institutional ethics committee of the First Affiliated Hospital of Anhui Medical University, and written informed consent was obtained from all participants in accordance with the Declaration of Helsinki.

### Preparation of Lysates for Proteomics

We collected 10ml of peripheral blood from individuals in cohort 1. Peripheral blood mononuclear cell (PBMC) was isolated with density-gradient centrifugation, then washed twice with ice-cold PBS and lysed in fresh lysis buffer consisting of 0.1M tetraethylammonium bromide (TEAB) (Thermo Fisher Scientific, MA, USA), 0.5% sodium dodecyl sulfate (SDS) (ST628, Beyotime, China), and 1X HALT™ protease and phosphatase inhibitor cocktail (78420, Thermo Fisher Scientific, MA, USA) (200µL) for 10 min. Protein concentration of the lysate was quantified by the BCA protein assay according to the manufacturer’s instructions (A53225, Thermo Fisher Scientific, MA, USA). Protein integrity was assessed by SDS-polyacrylamide gel electrophoresis (PAGE).

### Mass Spectrometry and Data Analyses

Detailed analyses were described as previously (17). In brief, Peptides were separated on an EASY-Spray C18 column (75 μm x 50cm inner diameter, 2 μm particle size, and 100 Å pore size, Thermo Fisher Scientific). Peptide fractions were gradient from 4%-22% solvent B (100% acetonitrile and 0.1% formic acid) over 70min, 22%-30% solvent B over 14min, 30%-80% solvent B over 3min, and 80% solvent B over 3min at a rate of 450 nL/min. An electrospray voltage of 2.0 kV was applied to the eluent *via* the EASY-Spray column electrode. the Lumos was operated in positive ion data-dependent mode, using Synchronous Precursor Selection (SPS-MS3) 7. Full scan MS1 was performed in the Orbitrap with a precursor selection range of 100–1700 m/z at a nominal resolution of 17500. The AGC was set to 4 x 10^5^, then MS2/MS3 analysis was conducted with the top ten precursors. Mass filtering was performed by the quadrupole with 0.7 m/z transmission window, followed by CID fragmentation in the linear ion trap with 35% normalized collision energy in rapid scan mode and a parallelizable time option was selected. SPS was applied to co-select 10 fragment ions for HCD-MS3 analysis.

For quality control of the expression data, we filtered low abundant proteins (< 1 in > 80% of samples) and converted expression data to logarithm form, which meets the normal distribution. Limma package (v.3.50.1) (18) was applied to define differentially expressed proteins (DEPs) between two groups with a 1.5-fold change and p-value less than 0.05. Dimension reduction and visualization of data were generated using Uniform Manifold Approximation and Projection (UMAP) with n_neighbors = 8 and min_dist=0. Gene Ontology (GO) and Kyoto Encyclopedia Genes and Genomes (KEGG) pathway enrichment analyses were conducted respectively using over-representation analysis implemented in the ClusterProfiler R package (v.3.14.3) (19). Hallmark gene set enrichment analysis was performed using GSEA (v.4.1.0) (20, 21). To find modules (highly correlated proteins) that are related to clinical information, we employed the WGCNA R package (v.1.70-3) (22) to construct a signed network with soft-thresholding powers of 4, minimum module size of 30, and cut height for merging of modules of 0.30. Pearson test was used to test for the association of modules with SLE characteristics.

### Western Blot

We collected 5 ml of peripheral blood from individuals in cohort 2. PBMCs were isolated with density-gradient centrifugation, then washed twice and lysed in RIPA buffer (Sigma-Aldrich, MO, USA) supplemented with protease and phosphatase inhibitor cocktail (Thermo Fisher Scientific, MA, USA). Protein concentration of lysates was determined using the BCA Protein Assay Kit according to the manufacturers’ instructions (A53225, Thermo Fisher Scientific, MA, USA). Cell lysates were boiled for 10 min at 95°C with SDS and subjected to 12% SDS–PAGE, and then transferred to nitrocellulose membranes (HATF00010, Millipore). The membranes were blocked and then incubated with anti-ELANE (ab131260, Abcam), anti-CD14 (DF13278, Abcam), anti-S100A11(ab169530, Abcam), anti-PGAM1(DF12693, affinity), anti-SERPINB10(DF9894, affinity), anti-BST2 (DF3846, affinity), and anti-β-actin (AF7018, affinity) overnight at 4°C. The membranes were washed and incubated with anti-rabbit or -mouse IgG-HRP (S0001, affinity) for 1 h. Protein bands were visualized with the western blotting detection system Tanon-5200 (Bio-Tanon, China). Gray value analysis was done by ImageJ (v.1.50g, NIH) software.

### Measurement of Biomarkers in Plasma Samples

We collected 5ml of peripheral blood from individuals in cohort 3, The cells are removed by centrifugation 3000g for 5min. The supernatant, designated plasma is carefully collected from the cell pellet using a Pasteur pipette. Levels of calprotectin (S100A8/A9) and glutathione in plasma samples were analyzed using a commercial enzyme-linked immunosorbent assay (ELISA) (ab267628, Abcam, USA) kit and Micro Reduced Glutathione (GSH) Assay Kit (BC1175, Solarbio, Beijing) respectively. Then elastase and cell-free DNA (cfDNA) were included for detecting NET (23). The level of Elastase was quantified *via* ELISA (ab119553, Abcam, USA). For the detection of cell-free DNA (cfDNA), we used PicoGreen dsDNA Assays Kits (P7589, Thermo Fisher Scientific, China) to quantify the cfDNA, the protocol is as follows: 1. Dilute the concentrated PicoGreen^®^ Dye stock two-hundred (200) fold with 1×TE; 2. Prepare Standard Curve with 1mg Deoxyribonucleic acid from calf thymus Type XV(D4522-1MG, Sigma, USA); 3. Measurement of fluorescence *via* Modulus Luminometer (9200-003, Turner BioSystems) and calculate cfDNA concentrations.

### Statistical Analysis

Shapiro-Wilk test of normality was performed using R (v.3.6.0, https://www.r-project.org/). All continuous variables conform to a normal distribution are expressed as Mean ± SD. All categorical variables are expressed as number and percentage of counts. The statistical significance was determined by unpaired two-tailed Student’s t-test for two-group comparisons and by one-way ANOVA followed by Bonferroni’s multiple-comparisons test for multi-group comparisons using R (v.3.6.0, https://www.r-project.org/). P values of < 0.05 were considered statistically significant.

## Results

### Diversity in Protein Abundance Between Sex and Disease Status

A total of 15 HC and 15 SLE (females: n=8/group, males: n=7/group) were included for proteomic analysis. We applied a stringent quality control to remove low abundant proteins (in methods) and included male SLE and female SLE with a comparable phenotype including age, SLEDAI scores, proteinuria, and the proportion of anti-dsDNA antibody (**Supplementary Table 1**), which may otherwise bias our results.

**Figure 1:**
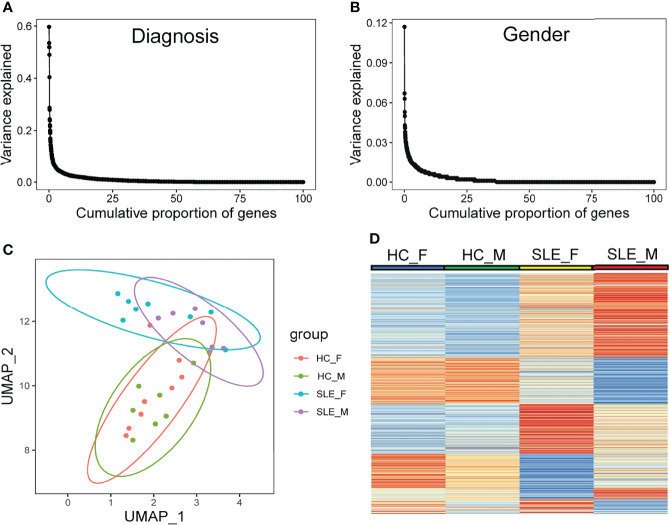
Diversity in protein abundance among SLE and healthy control with different gender. **(A, B)** Proportion of gene-expression variance explained by diagnosis and gender. **(C)** UMAP plot of all identified proteins. **(D)** Heatmap plot showing differentially expressed proteins in any comparison group among male HC, male SLE, female HC, and female HC. HC_F, female healthy control; HC_M, male healthy control; SLE_F, female SLE; SLE_M, male SLE.

Using mass spectrometry analysis, we identified a total of 4830 proteins, of which 383 (7.9%) and 768 (15.9%) were detected only in male SLE and female SLE compared to male and female HC, respectively. With a random-effects model, clinical diagnosis (SLE and non-SLE) and gender differences explained non-zero variance of gene expression for approximately 25% of genes, and clinical diagnosis explained a higher variance of expression than gender differences (**Figures 1A, B**). Furthermore, Uniform Manifold Approximation and Projection for Dimension Reduction (UMAP) and heatmap of differentially expressed proteins (DEPs) demonstrated that male SLE, female SLE, male control, and female control have a heterogeneous proteomic profile (**Figures 1C, D**), indicating that proteomic studies with different gender samples could help to reveal the pathogenesis of SLE in different genders.

### Weighted Gene Co-Expression Network Analysis Highlights Important Gene Modules

**Figure 2:**
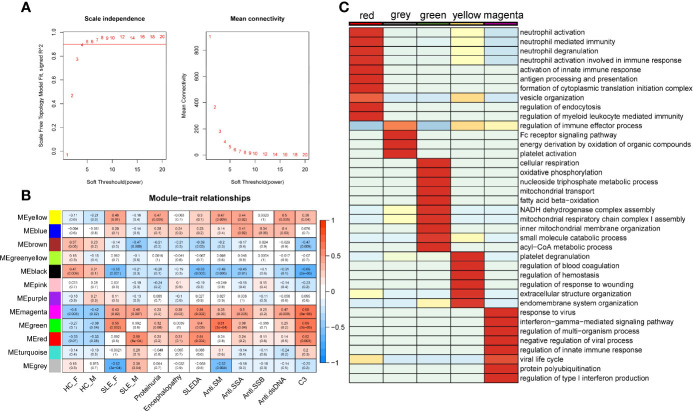
SLE-associated modules and their functional meaning. **(A)** Summary network indices (y-axes) as functions of the soft thresholding power. **(B)** Relationships of consensus module eigengenes and clinical traits. Numbers in the table report the correlations of the corresponding module eigengenes and traits, with the p-values printed below the correlations in parentheses. **(C)** Heatmap comparing enrichment p-value of pathway among five modules. The stronger the red color, the more significant the p-value is.

Weighted gene co-expression network analysis (WGCNA) is a systematic biology method for describing the correlation patterns (modules) among highly correlated genes, suggesting genes with coordinated changes in expression are more likely to be involved in similar biological significance. One of the advantages of this approach is that WGCNA can use all gene expression information to calculate modules and further relate modules to external sample traits. Here, we applied WGCNA to generate a network from the PMBC protein profiles of 30 individuals (22). We chose soft-thresholding powers = 4 based on the criterion of approximate scale-free topology (**Figure 2A**), which identified 12 modules of highly correlated proteins, represented by different colors. We further related these modules to external disease diagnosis as well as gender information (male SLE, female SLE, male HC and female HC, **Figure 2B**), and found that red (r = 0.58, *p* = 5×10^−04^) and grey (r=0.38, *p* = 0.04) modules are positively correlated with male SLE, while yellow (r=0.48, *p*=0.01) and green (r=0.55, *p*=0.002) modules are positively correlated with female SLE. In addition, the magenta module is positively correlated with both male SLE (r=0.48, *p*=0.007) and female SLE (r=0.43, *p*=0.02). Subsequently, we performed gene-ontology biological processes analysis and found that proteins in red and grey modules (positively correlated with male SLE) were mapped to known signaling pathways including neutrophil activation networks, Fc receptor signaling pathway, platelet activation, and antigen processing and presentation (**Supplementary Figures 1A, B**), while proteins in yellow and green modules (positively correlated with female SLE) were mapped to cellular respiration, oxidative phosphorylation, and neutrophil activation networks (**Supplementary Figures 1D, E**). Notably, proteins in the magenta modules (positively correlated with both male SLE and female SLE) were mapped to type I IFN production and response to virus (**Supplementary Figure 1C**). To further compare the significance of biological processes among five modules, we generated a heatmap (**Figure 2C**), which shows the red module was most enriched in neutrophil activation works, while yellow and grey modules mapped to neutrophil activation works with a moderate significance. Consistently, the magenta module was most enriched in IFN signaling and the green module was most enriched in metabolism pathways including cellular respiration and oxidative phosphorylation. Taken together, type I IFN signaling and neutrophil activation networks mapped to both male and female SLE, while cellular respiration and oxidative phosphorylation only mapped to female SLE.

We also related modules to external clinical traits including proteinuria, systemic lupus erythematosus disease activity index (SLEDAI), and anti-dsDNA antibody (**Figure 2B**). The yellow module is positively correlated with proteinuria (r = 0.47, *p* = 0.009), indicating that neutrophil activation networks may be involved in renal damage. The yellow and magenta module that positively correlated with anti-dsDNA, indicating type I IFN and neutrophil activation networks promote the production of autoantibody. The magenta, green and red modules are positively correlated with SLEDAI and C3 levels, suggesting type I IFN response, neutrophil activation networks, and oxidative phosphorylation all contribute to SLE activity (**Supplementary Figure 1**).

### Differential Protein Analysis Revealed Shared Pathogenesis and Gender-Specific Pathogenesis of SLE

**Figure 3:**
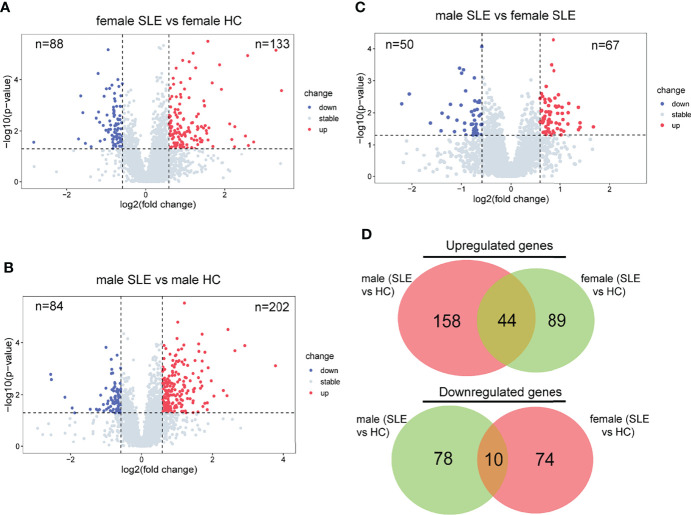
Proteomic heterogeneity among SLE and healthy control with different gender. **(A–C)** Volcano plot showing DEPs between female SLE versus female HC, male SLE versus male HC, and male SLE versus female SLE. **(D)** Venn diagram showing number of proteins with significant upregulation or downregulation comparing SLE with healthy control.

To further decompose the pathogenesis of SLE, we performed differential protein analyses of three binary comparison groups: female SLE versus female HC; male SLE versus male HC; male SLE versus female SLE. Indeed, 133 proteins were more abundant and 88 proteins were less abundant at the proteomics level in female SLE compared with female HC (**Figure 3A**), while 202 proteins were more abundant and 84 proteins were less abundant in male SLE compared with male HC (fold change > 1.5 or <0.67; *p* < 0.05, **Figure 3**). In contrast, male SLE and female SLE showed a similar proteomic profile, with only 67 proteins up-regulated and 50 proteins down-regulated in male SLE compared with female SLE (**Figure 3C**). Strikingly, DEPs in male SLE versus male HC were highly distinct from those in female SLE versus female HC, only a proportion of DEPs was common in the two comparison groups (**Figure 3D**), suggesting heterogeneity may exist in the pathogenesis of male SLE and female SLE.

**Figure 4:**
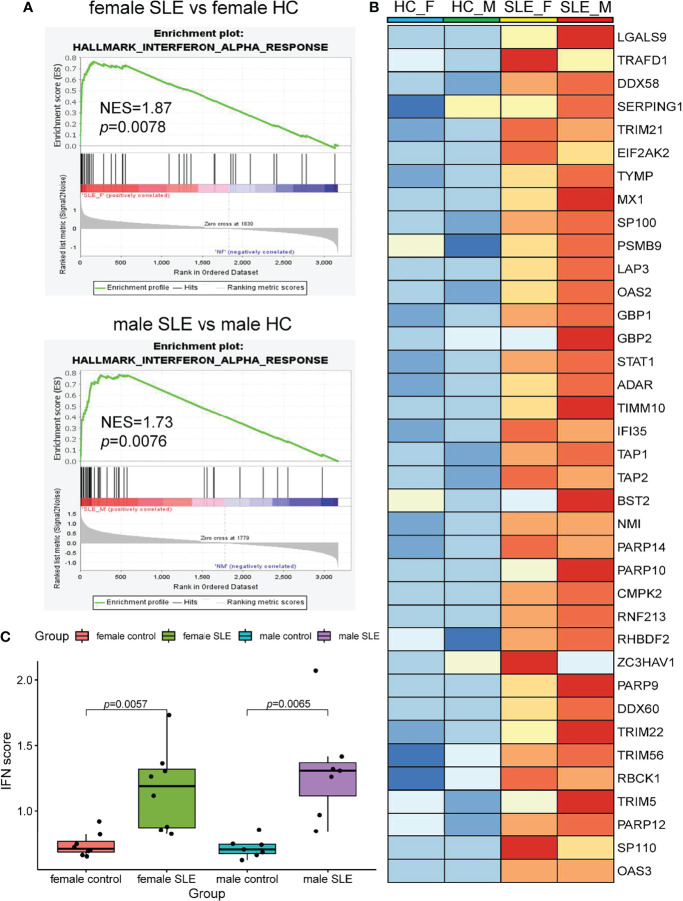
Interferon signaling activation is a common feature in male SLE and female SLE. **(A)** Hallmarks gene set enrichment analysis (GSEA) showing pathways enriched in SLE compared to healthy control with different gender. NES: normalized enrichment score, p: p-value of enrichment analysis. **(B)** Boxplot showing type I interferon score among SLE and healthy control with different gender. **(C)** Heatmap showing the expression of core proteins that contribute to type I interferon pathway enrichment (red, high abundance; blue, low abundance).

It is well documented that SLE patients display elevated type I IFN-stimulated genes (ISGs) in multiple cells and organs including peripheral blood mononuclear cells (PBMCs), low-density granulocytes (LDG), skin, and kidney (9, 17, 24, 25). To determine whether SLE PBMC also displays increased ISG expression at the proteomic level, we performed a GSEA of up-regulated proteins in female SLE and male SLE relative to female HC and male HC respectively. As expected, our proteomic analysis showed that type I IFN signaling pathway is up-regulated in both male SLE and female SLE (**Figure 4A, B**). We further included a panel of 109 ISGs (54 were detected in our data) to construct an IFN score, as described previously (26). Indeed, IFN scores were significantly higher in both female SLE and male SLE relative to female HC (*p* = 0.0057) and male HC (*p* = 0.0065). while no significant difference between male SLE and female SLE (*p* > 0.05, **Figure 4C**). These results verified the aforementioned WGCNA findings that magenta module, the shared module positively correlated with both male SLE and female SLE, is most enriched in type I IFN signaling. Therefore, our data provide strong evidence that high type I IFN activity is a common feature in both male SLE and female SLE.

**Figure 5:**
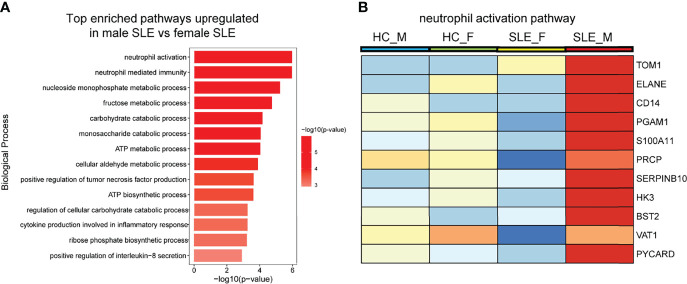
Neutrophil activation works is more prominent in male SLE relative to female SLE. **(A)** Bar plot showing top enriched pathways upregulated in male SLE versus female SLE. **(B)** Heatmap showing the expression of core proteins that contribute to neutrophil activation pathway enrichment (red, high abundance; blue, low abundance). HC_F, female healthy control; HC_M, male healthy control; SLE_F, female SLE; SLE_M, male SLE.

Enrichment analysis also revealed that proteins more abundant in male SLE relative to male HC were most enriched in neutrophil activation networks (**Supplementary Figure 2A**). However, proteins more abundant in female SLE relative to female HC were not mapped to neutrophil activation networks and oxidative phosphorylation pathway (**Supplementary Figure 2B**), inconsistent with WGCNA finding that proteins in the yellow and green module (positively correlated with female SLE) were enriched in neutrophil activation networks and oxidative phosphorylation. We suspect that a subset of weaker up-regulated proteins in female SLE enriched in these two pathways. Thus, we selected up-regulated proteins in female SLE (fold change > 1.2 and <1.5) relative to female HC and re-conducted gene-ontology biological processes enrichment analysis, in which we detected these proteins mapping to neutrophil activation networks and oxidative phosphorylation pathway (**Supplementary Figure 2C**). Finally, we directly compared male SLE with female SLE and found that proteins more abundant in male SLE also mapped to neutrophil activation networks (**Figures 5A, B**). These findings verified that although neutrophil activation networks were up-regulated in both female SLE and male SLE, the level of neutrophil activation is significantly higher in male SLE compared with female SLE.

### Western Blot Validation of Proteins in Neutrophil Activation Networks

**Figure 6:**
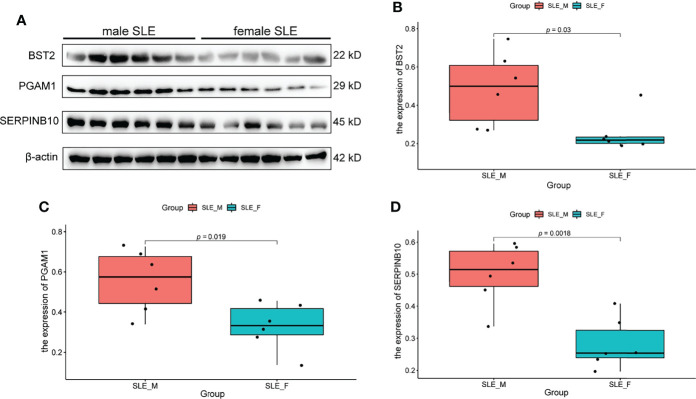
Western Blot Validation of Proteins in Neutrophil Activation Networks. **(A)** Western blot analysis of BST2, PGAM1 and SERPINB10 in male SLE and female SLE. **(B)** Boxplot showing gray value of BST2 in male SLE and female SLE. **(C)** Boxplot showing gray value of PGAM1 in male SLE and female SLE. **(D)** Boxplot showing gray value of SERPINB10 in male SLE and female SLE.

Based on the fold change (fold change > 1.8 in male SLE compared with female SLE) and P value (*p* < 0.05), we selected the top 6 proteins (ELANE, CD14, PGAM1, S100A11, SERPINB10, and BST2) that involved in neutrophil activation and performed western blot to verify our proteomic finding. According to the Western blotting analysis, we found that the relative expression levels of PGAM1, BST2, and SERPINB10 were significantly increased in male SLE compared with female SLE (*p* < 0.05). Although not reaching statistical significance, ELANE and S100A11 were also more abundantly expressed in male SLE relative to female SLE (**Figure 6**).

### Biomarkers of Neutrophil Activation Is Higher in Male SLE

**Figure 7:**
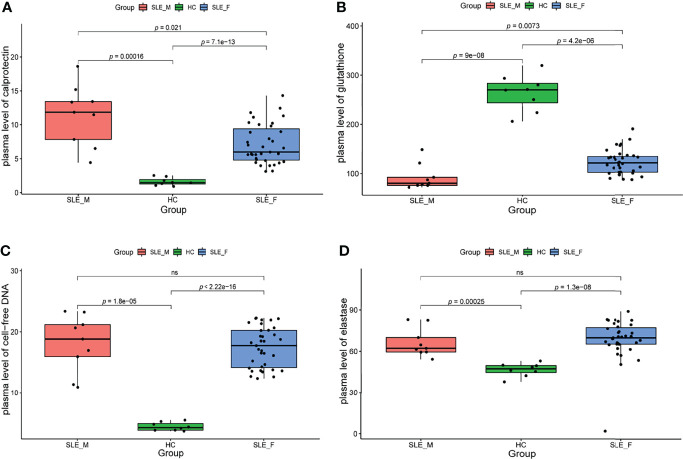
The level of neutrophil activation and oxidative stress is higher in male SLE. **(A)** Boxplot showing serum level of calprotectin in male SLE, female SLE, and healthy control. **(B)** Boxplot showing serum level of glutathione in male SLE, female SLE, and healthy control. **(C)** Boxplot showing serum level of cell-free DNA in male SLE, female SLE, and healthy control. **(D)** Boxplot showing serum level of elastase in male SLE, female SLE, and healthy control. ns, not significant.

We further determined the biomarkers of neutrophil activation (calprotectin), NETosis (cfDNA and elastase) (27), and ROS (glutathione) (28) in an independent sample cohort (n=52, **Supplementary Table 2**). To exclude the effect of confounding factors such as disease activity, we included male SLE and female SLE with comparable age, SLEDAI scores, proteinuria, and the proportion of anti-dsDNA antibody. In contrast to healthy control, both male and female SLE have a higher level of calprotectin, cfDNA, elastase, and ROS (low glutathione), suggesting exaggerated neutrophil activation participates in SLE through the production of reactive oxygen species (ROS) and the formation of neutrophil extracellular traps (NETs). More importantly, male SLE has a higher level of calprotectin and ROS (lower level of glutathione) in relative to female SLE (**Figure 7**). These results verified that male SLE has a higher level of neutrophil activation, which may promote rapid disease progression by releasing large amounts of ROS.

## Discussion

To our knowledge, ours is the first study to explore the gender heterogeneity in the pathogenesis of SLE at the protein expression level. Based on the bioinformatic analysis and biomarkers validation, we found that type I IFN activation is a common pathogenic pathway in male and female SLE, while neutrophil activation is more prominent in male SLE relative to female SLE. Here, we were able to successfully decompose the pathogenesis of SLE into a shared component and a gender-specific component.

Over the past decade, an increasing number of omics studies have been conducted in SLE cohorts (29). However, most of the studies have been conducted at the transcriptional level and focused primarily on females. These studies have repeatedly identified the activation of type I IFN signaling as manifested by upregulation of ISG in multiple cells and organs (9, 17, 24, 25). Consistently, our proteomic analysis of PBMC revealed that ISG levels were significantly higher in female SLE relative to female HC. In addition, we revealed that ISGs were also up-regulated in male SLE and that IFN scores were comparable in male SLE and female SLE. Therefore, our findings at the proteomic level demonstrate that type I IFN activation is a common feature in male SLE and female SLE.

Neutrophil activation and the formation of neutrophil extracellular traps (NETs) are hallmarks of innate immune activation. Recently, studies have shown that neutrophil dysregulation is implicated in the pathogenesis of SLE (9, 17). Neutrophils are viewed as a heterogeneous cell population, including normal density neutrophils (NDNs) and low-density granulocytes (LDGs). The two neutrophil subsets, especially LDGs are more likely to activate and form neutrophil extracellular traps (NETs), contributing to the cycle of inflammation. Of note, after density-gradient centrifugation, the neutrophils were in the bottom layer with red blood cells while LDGs are present in PBMC layer. Indeed, early in 2003, Bennett et al. (8) discovered a high expression of neutrophil-specific genes in pediatric SLE patients, and this “granulocyte signature” was due to the increase of LDGs in the PBMC layer. Thus, our findings of high levels of neutrophil activation, especially in male SLE, may be the result of LDG activation. Previous study also reported that LDG activation and their formed NETs could induce endothelial and organ damage including cardiovascular disease and lupus nephritis (30). In our study, we demonstrated that male SLE has a higher level of neutrophil activation signaling than female SLE, which may explain why SLE is more likely to cause organ damage in men relative to women. In addition, oxidative stress is increased in SLE, which contributes to immune system dysregulation, abnormal activation of cell death signals and autoantibody production (31). It is reported that ROS, products of oxidative stress released by neutrophils, are key signaling molecules that cause inflammation and organ damage in SLE and inflammatory diseases (28, 32, 33). Our study revealed a decreased glutathione (representing an increase in ROS) in SLE relative to healthy control. Moreover, male SLE presents with a lower level of glutathione (an increase level of ROS) and a higher level of the inflammatory pathways (i.e. TNF production; **Figure 5A**) than female SLE. These results suggest that stronger neutrophil activation in male SLE may promote rapid disease progression and organ damage by releasing large amounts of ROS.

Overall, our results decompose the pathogenesis of SLE into a shared component and a gender-specific component. Male SLE has the highest level of neutrophil activation accounts for the most significant associations with organ damage and poor prognosis. This study adds to our understanding of gender heterogeneity in the pathogenesis of SLE, an area that lagged far behind compared to clinical heterogeneity. Future studies should reveal why neutrophil activation is higher in male SLE and the key molecules through which the pathway functions. Furthermore, this study suggests that specifically targeting key markers in neutrophil activation networks may play important role in the treatment of male SLE.

## Data Availability Statement

The datasets presented in this study can be found in online repositories. The name of the repository and accession number can be found below: Proteome Xchange (http://proteomecentral.proteomexchange.org); PXD033144.

## Ethics Statement

The studies involving human participants were reviewed and approved by the institutional ethics committee of the First Affiliated Hospital of Anhui Medical University. The patients/participants provided their written informed consent to participate in this study.

## Author Contributions

Conceptualization: MC, LG, and YS. Formal analysis and visualization: MC and LG. Data curation: LZ, HH, and YZ. Writing - original draft preparation: MC and LG. Investigation: MC and HH. Writing - review and editing: YS and ZC. Funding acquisition: YS. All authors have read and agreed to the published version of the manuscript. All authors contributed to the article and approved the submitted version.

## Funding

This study was funded by the National Natural Science Foundation of China (81872527, 81830019).

## Conflict of Interest

The authors declare that the research was conducted in the absence of any commercial or financial relationships that could be construed as a potential conflict of interest.

## Publisher’s Note

All claims expressed in this article are solely those of the authors and do not necessarily represent those of their affiliated organizations, or those of the publisher, the editors and the reviewers. Any product that may be evaluated in this article, or claim that may be made by its manufacturer, is not guaranteed or endorsed by the publisher.
