# Supplementary figures and images for: Proteomic Analyses Reveal Higher Levels of Neutrophil Activation in Men Than in Women With Systemic Lupus Erythematosus
Source: Front Immunol. 2022 Jun 21;13:911997. doi: 10.3389/fimmu.2022.911997 (PMC9254905; doi:10.3389/fimmu.2022.911997)

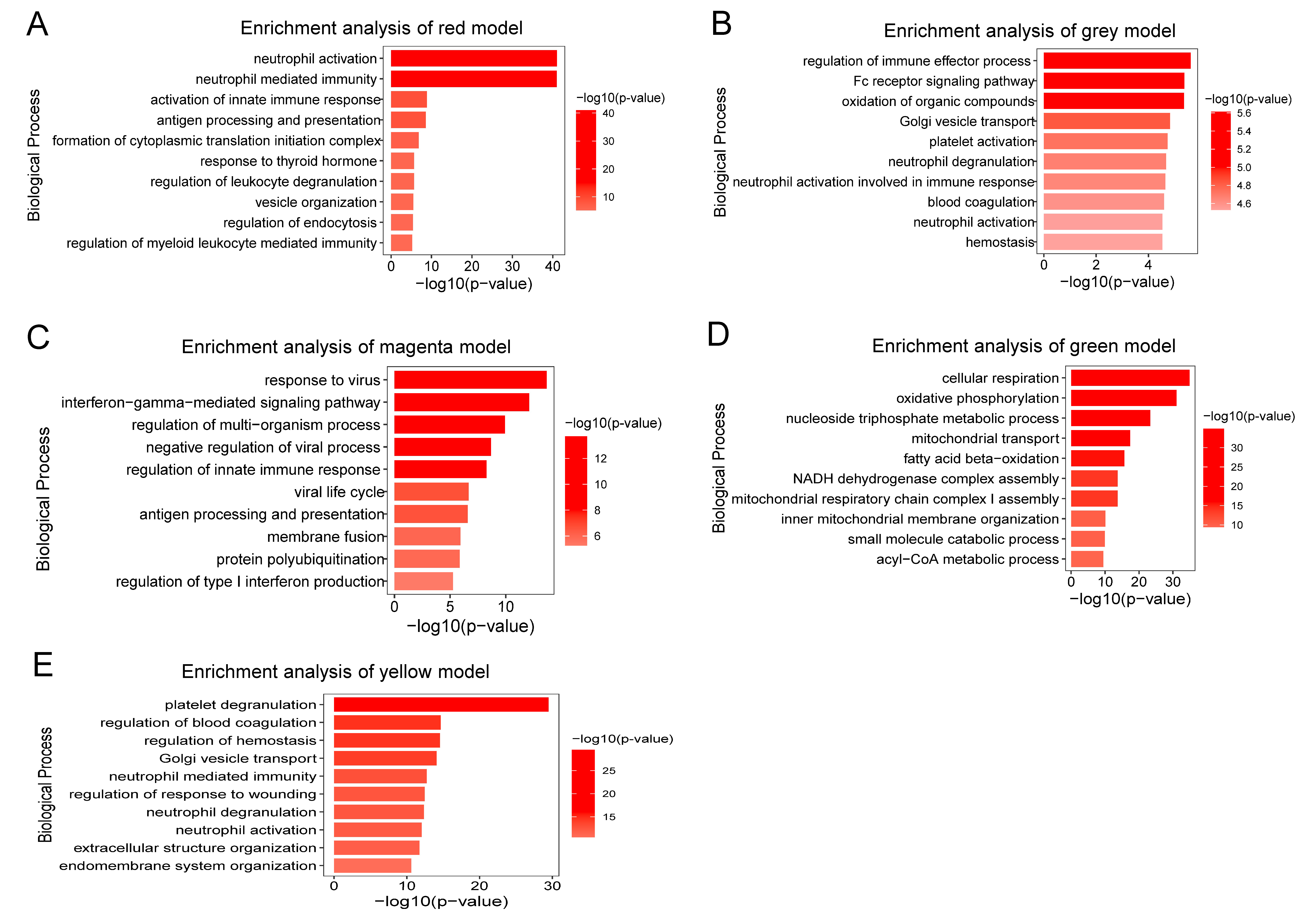

Supplement: Supplementary file 1 [file Image_1.tif]

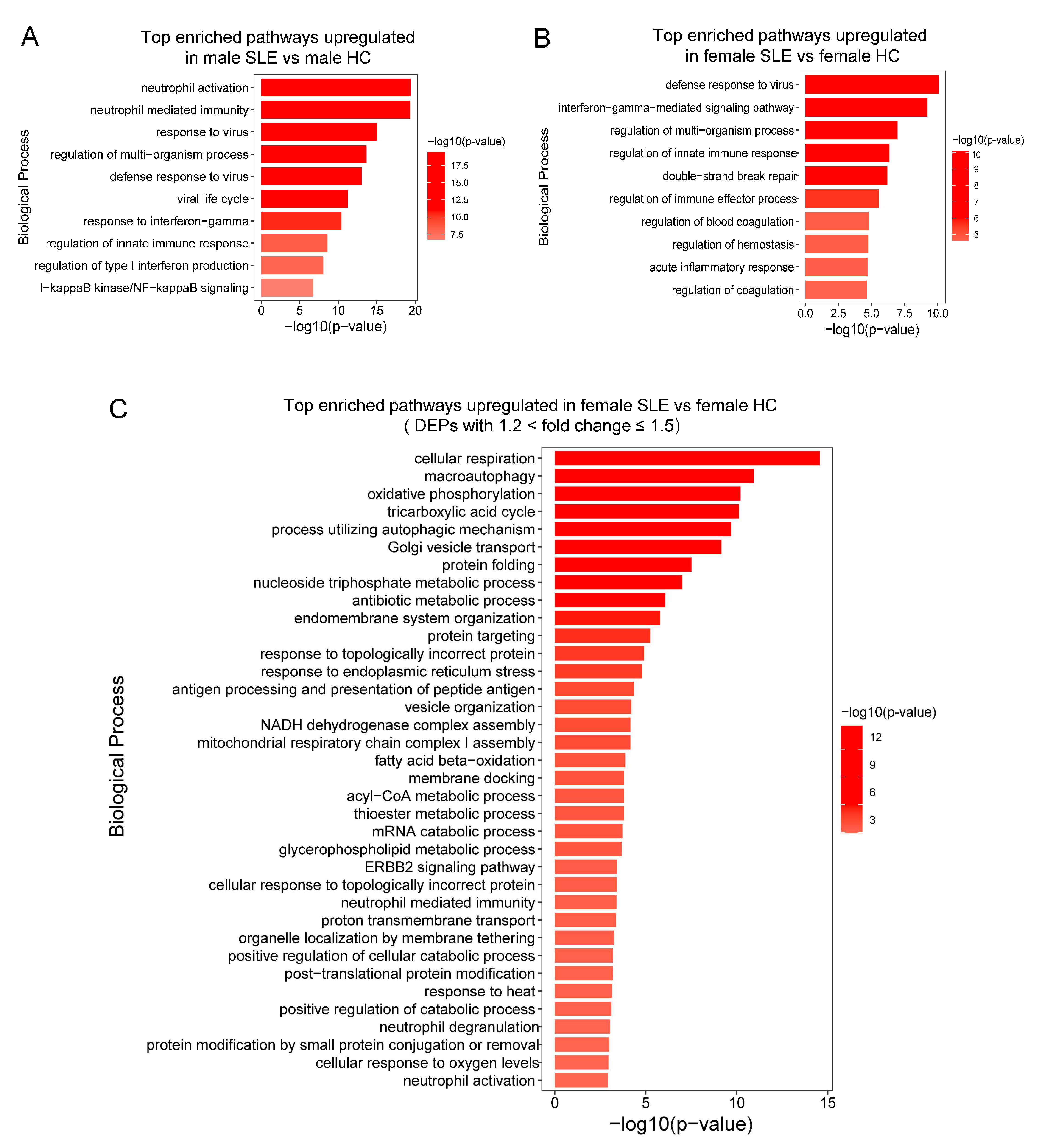

Supplement: Supplementary file 2 [file Image_2.tif]

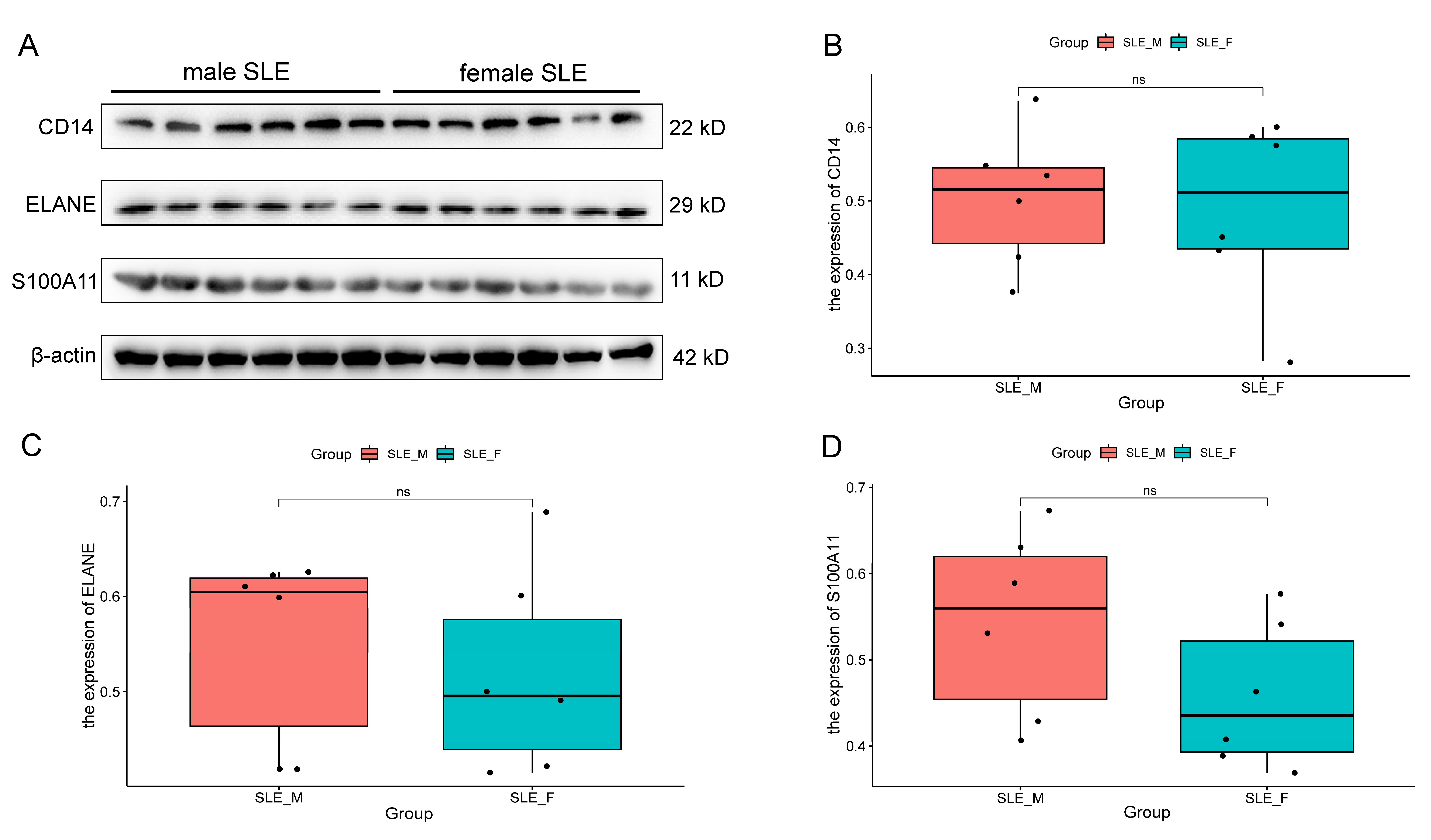

Supplement: Supplementary file 3 [file Image_3.tif]
