# Supplementary material for: Proteomic Analyses Reveal Higher Levels of Neutrophil Activation in Men Than in Women With Systemic Lupus Erythematosus
Source: Front Immunol. 2022 Jun 21;13:911997. doi: 10.3389/fimmu.2022.911997 (PMC9254905; doi:10.3389/fimmu.2022.911997)
Supplement: Supplementary file 4 [file Table_1.docx]

Supplementary Material

# Supplementary Figures and Tables

## Supplementary Figures

**Supplementary Figure 1.** Biological process enrichment analysis of proteins in modules. (A-E) Bar plot showing top enriched pathways in red, grey, magenta, green, and yellow modules.

**Supplementary Figure 2.** Differentially expressed proteins in male SLE and female SLE mapped to different pathway. (A-B) Bar plot showing top enriched pathways upregulated in male SLE versus male HC and female SLE versus female HC (DEPs defined as fold change > 1.5 and p-value < 0.05). (C) Bar plot showing top enriched pathways upregulated in female SLE versus female HC (DEPs defined as 1.2 < fold change < 1.5 and p-value < 0.05)

**Supplementary Figure 3.** Western Blot Validation of Proteins in Neutrophil Activation Networks. (A) Western blot analysis of CD14, ELANE, and S100A11 in male SLE and female SLE. (B) Boxplot showing gray value of CD14 in male SLE and female SLE. (C) Boxplot showing gray value of ELANE in male SLE and female SLE. (D) Boxplot showing gray value of S100A11 in male SLE and female SLE.

## Supplementary Tables

Supplementary Table 1. Protein expression of SLE and HCs in cohort 1.

Supplementary Table 2. Characteristics of HCs and patients with SLE in cohort 1.

**Supplementary Table 3.** plasma biomarkers of HCs and patients with SLE in cohort 3.
