# Supplementary material for: Proteomic Analyses Reveal Higher Levels of Neutrophil Activation in Men Than in Women With Systemic Lupus Erythematosus
Source: Front Immunol. 2022 Jun 21;13:911997. doi: 10.3389/fimmu.2022.911997 (PMC9254905; doi:10.3389/fimmu.2022.911997)
Supplement: Supplementary file 6 [file Table_3.docx]

**Supplement Table 2. Characteristics of HCs and patients with SLE in cohort 1**

| Characteristics | HC | Male SLE | Female SLE | *p* |
| --- | --- | --- | --- | --- |
| Individuals, n | 15 | 7 | 8 | - |
| Age, year (mean±SD) | 33.53±6.47 | 35.43±8.73 | 33.75±8.11 | 0.71 |
| Age onset, year (mean±SD) | - | 31.14±10.64 | 28.50±4.53 | 0.56 |
| SLEDAI (mean±SD) | - | 7.85±5.61 | 5.13±3.14 | 0.28 |
| Proteinuria (>0.5g/24h), n, % | - | 3, 42.85% | 4, 50% | 1 |
| Malar erythema, n, % | - | 5, 66.67% | 3, 37.5% | 0.81 |
| Photaesthesia, n, % | - | 2, 28.57% | 2, 25% | 1 |
| Mucosal ulcers, n, % | - | 0, 0% | 1, 12.5% | - |
| Arthritis, n, % | - | 1, 14.29% | 1, 12.5% | - |
| Pleurisy, n, % | - | 0, 0% | 0, 0% | - |
| Psychosis, n, % | - | 2, 28.57% | 0, 0% | - |
| Fever, n, % | - | 0(0%) | 0(0%) | - |
| Lupus encephalopathy, n, % | - | 3, 42.85% | 1, 12.5% | 0.46 |
| Alopecia | - | 1, 14.29% | 1, 12.5% | - |
| ANA, n, % | - | 7, 100% | 8, 100% | - |
| Anti-JO-1 antibody, n, % | - | 0, 0% | 1, 12.5% | - |
| Anti-SM antibody, n, % | - | 0, 0% | 5, 62.5% | - |
| Anti-SSA antibody, n, % | - | 5, 71.4% | 5, 62.5% | 1 |
| Anti-SSB antibody, n, % | - | 1, 14.29% | 2, 25% | - |
| Low C3, n, % | - | 5, 71.42% | 7, 87.5% | 0.89 |
| Anti-dsDNA antibody, n, % | - | 3, 42.85% | 3, 37.5% | 1 |
