# Supplementary material for: Proteomic Analyses Reveal Higher Levels of Neutrophil Activation in Men Than in Women With Systemic Lupus Erythematosus
Source: Front Immunol. 2022 Jun 21;13:911997. doi: 10.3389/fimmu.2022.911997 (PMC9254905; doi:10.3389/fimmu.2022.911997)
Supplement: Supplementary file 7 [file Table_4.docx]

**Supplement Table3. plasma biomarkers of HCs and patients with SLE in cohort 3**

| Biomarkers/characteristics | HC | female SLE | male SLE | *p* value |
| --- | --- | --- | --- | --- |
| Age, year (mean±SD) | 28.13±4.99 | 30.83±7.71 | 30.67±8.87 | 0.66 |
| Proteinuria (>0.5g/24h), n, % | - | 17, 44.74% | 5, 31.25% | 0.54 |
| Anti-dsDNA, n, % | - | 20, 52.6% | 6, 37.5% | 0.47 |
| SLEDAI (mean±SD) | - | 7.43±4.69 | 6.67±4.21 | 0.64 |
| Glutathione (mean±SD) | 264.28±36.71 | 123.35±24.72 | 92.54±25.82 | < 2E-16 |
| Calprotectin (mean±SD) | 1.62±0.59 | 7.11±2.92 | 11.41±4.47 | 1.47E-07 |
| Elastase (mean±SD) | 46.59±4.79 | 68.61±14.66 | 66.33±10.24 | 3.53E-04 |
| cfDNA (mean±SD) | 4.42±0.68 | 17.46±3.28 | 18.04±4.66 | 5.54E-13 |
